# Impact of preoperative mean keratometry on corneal higher-order aberrations and optical quality following cataract surgery

**DOI:** 10.3389/fmed.2026.1934290

**Published:** 2026-10-13

**Authors:** Zhan Shen, Kaiyue Du, Bowei Yuan, Yinan Liu, Xiaoyong Chen, Jing Hong

**Affiliations:** Department of Ophthalmology, Peking University Third Hospital, Beijing, China

**Keywords:** cataract surgery, corneal curvature, higher-order aberrations, phacoemulsification, surgically induced astigmatism

## Abstract

**Purpose:**

To evaluate how different corneal meridian characteristics influence visual outcomes, surgically induced astigmatism (SIA), and higher-order aberrations (HOAs) following phacoemulsification with intraocular lens implantation in cataract patients.

**Methods:**

Patients were divided into three groups according to preoperative mean keratometry (Kmean): flat (≤ 43.0 D), regular (43.0–46.0 D), and steep (≥ 46.0 D). Outcomes included corrected distance visual acuity (CDVA), anterior and posterior corneal astigmatism, SIA measured with the Pentacam, and HOAs assessed using both Pentacam and iTrace at 1 and 3 months postoperatively.

**Results:**

CDVA, corneal astigmatism, and SIA parameters showed no significant between-group differences (all *p* > 0.05), with the primary clinically relevant differences lying in intrinsic HOAs and aberration changes. Preoperatively, Pentacam measurements revealed that the steep group had significantly higher anterior and posterior corneal HOAs than the flat group (*p* = 0.006 and <0.001, respectively). At 3 months postoperatively, the steep group maintained higher HOAs (*p* = 0.006 and 0.013), while changes from baseline were comparable among groups. iTrace analysis showed that the flat group had the lowest preoperative spherical aberration (SA) (*p* = 0.003), but exhibited a significant postoperative increase in trefoil aberration compared with the regular group (mean difference, 0.10 μm; 95% CI, 0.02 to 0.18; *P*adj=0.042), alongside a non-significant trend toward higher SA.

**Conclusion:**

Cataract surgery provides effective and consistent refractive and visual outcomes in eyes across different Kmean profiles. Steeper corneas consistently show higher intrinsic HOAs, whereas flatter corneas demonstrate a statistically significant increase in trefoil and a non-significant trend toward higher SA compared with regular corneas.

## Introduction

Cataracts are the leading cause of reversible blindness worldwide, making cataract surgery the most frequently performed surgical procedure. Over recent decades, cataract surgery has progressed from a primarily rehabilitative intervention to a refractive procedure designed to optimize postoperative visual quality (1). Within this context, surgically induced astigmatism (SIA) and higher-order aberrations (HOAs) have become critical determinants of patient satisfaction. Correction of preexisting astigmatism is essential for achieving early visual rehabilitation and emmetropia, while minimizing HOAs is equally important for preserving optical quality (2, 3).

Previous studies have reported significant increases in corneal HOAs following cataract surgery (4, 5). The extent of SIA and HOAs is influenced by modifiable surgical parameters such as incision size, location, configuration, and tunnel length (6, 7), as well as ocular characteristics that cannot be controlled, including laterality (8), corneal thickness (9), corneal diameter (10), and the magnitude and type of preoperative astigmatism (11).

Corneal meridian configuration has been shown to affect preoperative corneal HOAs (12). Furthermore, studies by Mostafa et al.and Xu et al. (13, 14) have reported that eyes with extreme keratometry—specifically flat (≤ 43.0 D) or steep (≥ 46.0 D) profiles—present unique clinical challenges, significantly influencing refractive outcomes and the accuracy of intraocular lens calculation formulas. With the increasing demand for optimal postoperative visual quality, understanding the isolated optical properties of the cornea has become paramount. In the phakic eye, the crystalline lens partially compensates for the cornea's positive spherical aberration to optimize retinal image quality (15, 16). However, cataract extraction permanently abolishes this internal compensation mechanism, thereby fully unmasking the cornea's inherent higher-order aberrations (HOAs) (16). Consequently, eyes with extreme corneal meridian configurations—specifically flat or steep keratometry—often exhibit higher intrinsic HOAs and are more vulnerable to postoperative optical degradation (12). Furthermore, these extreme curvatures possess distinct biomechanical stress distributions, making them potentially more susceptible to incision-induced deformations, such as trefoil (12).

Additionally, as highlighted by Koch et al., relying on population-averaged models can introduce significant prediction errors in eyes that deviate from normal anatomical ranges (17). However, it remains uncertain whether these morphological differences influence postoperative astigmatism correction, the degree of SIA, or the induction of new HOAs. Evidence directly comparing postoperative visual quality across different corneal meridian types in real-world settings is limited. To bridge these knowledge gaps, the present prospective, real-world study was specifically designed to evaluate the impact of extreme corneal meridian configurations (Kmean stratification: flat, regular, and steep) on visual acuity, SIA, and both anterior/posterior and total HOAs—assessed via a combined Pentacam and iTrace protocol—following standard phacoemulsification and IOL implantation under routine clinical conditions. Our primary hypothesis was that extreme mean keratometry (flat or steep) is associated with distinct patterns of SIA and HOA induction following standardized phacoemulsification. Secondarily, we hypothesized that variations in corneal biomechanical properties render these extreme profiles differentially susceptible to specific intrinsic aberrations, such as incision-induced trefoil.

## Methods

### Participants

This prospective, real-world study included patients with age-related cataract undergoing phacoemulsification and IOL implantation between July 2024 and December 2024 at Peking University Third Hospital, Beijing, China. All participants provided informed consent. The study was approved by the hospital's ethics committee and performed in accordance with the tenets of the Declaration of Helsinki.

Consecutive patients with preoperative corneal astigmatism of less than 2.5 diopters (D) who strictly met the inclusion and exclusion criteria (as detailed in the patient screening flow chart in Supplementary Figure S1) were prospectively enrolled. Only the right eye of each participant was used in the study to eliminate inter-eye correlation bias, with preliminary assessments showing no systematic biometric differences between the right and left eyes. Enrolled patients were classified into three groups according to mean keratometry (Kmean) derived consistently from the Pentacam system: flat (≤ 43.0 D), regular (43.0–46.0 D), and steep (≥ 46.0 D) (13, 14); the distribution of Kmean across the entire cohort is detailed in Supplementary Figure S2. Exclusion criteria encompassed the presence of ocular surface disorders (such as severe dry eye, ectropion, entropion, or trichiasis), corneal disease, uveitis, glaucoma, retinopathy, neurological disorders, a history of ocular trauma or surgery, systemic diseases (including diabetes mellitus, thyroid disease, or autoimmune disorders), significant intraoperative or postoperative complications, and incomplete follow-up data.

### Clinical data measurement

All patients underwent a comprehensive ophthalmologic evaluation within two weeks before surgery. Preoperative assessments included uncorrected distance visual acuity, corrected distance visual acuity (CDVA), manifest and cycloplegic refraction, slit-lamp biomicroscopy, intraocular pressure measurement, endothelial cell density, ultrasound A- and B-scan, dilated indirect fundoscopy, anterior segment optical coherence tomography (Visante; Zeiss, Oberkochen, Germany), and ocular biometry using the IOL Master 700 (Zeiss, Jena, Germany). Additional examinations comprised corneal tomography (Pentacam; Oculus, Washington, DC, USA) and ray tracing wavefront aberrometry (iTrace; Tracey Technologies, Houston, TX, USA). All HOA measurements were performed under natural pupil conditions in a dimly lit room by a single experienced technician. A minimum of three consecutive scans were obtained for each eye using both the Pentacam and iTrace systems. For the Pentacam, only scans displaying an ‘OK’ Quality Specification (QS) were included (18). For the iTrace, scans were rigorously screened for proper centration, stable fixation, and the absence of Placido ring distortion to ensure strict quality control. The pupil diameter used for all HOA analyses was fixed at at a standardized 6.0 mm zone.

Preoperative markings for the tunnel and sideport incisions were made at the slit lamp while the patient was seated to minimize cyclotorsion in the supine position. SIA was calculated using vector analysis according to the Alpins method (19).

Postoperative follow-up was performed at 1 week, 1 month, and 3 months, and included visual acuity, manifest refraction, slit-lamp biomicroscopy, and intraocular pressure measurement. Pentacam imaging and iTrace aberrometry were performed at both the 1-month and 3-month visits.

### Surgical procedures

All surgical procedures were performed by a single experienced surgeon (the corresponding author). To standardize the biomechanical impact and prevent potential confounding effects of varying incision locations, a standardized 3.0 mm triplanar clear corneal main incision was consistently created superiorly at the 90-degree meridian, along with a 0.6 mm side-port incision. An ophthalmic viscosurgical device (OVD) was injected into the anterior chamber, and continuous curvilinear capsulorhexis was performed under navigation guidance, using a corneal plane diameter of 5.5–6.0 mm as the reference. Hydrodissection and hydrodelineation were subsequently performed. Cataract removal was achieved via phacoemulsification using the Centurion Vision System (Alcon Laboratories, Fort Worth, TX, USA). Patients with corneal astigmatism <1.0 D received monofocal IOLs, whereas those with 1.0–2.5 D astigmatism were implanted with toric IOLs. After reinjection of OVD into the anterior chamber, the selected IOL was implanted into the capsular bag and centered with the aid of the navigation system. Residual OVD was then removed, and the incisions were sealed by stromal hydration. At the conclusion of surgery, tobramycin–dexamethasone ointment was applied, and the operated eye was dressed with an eye pad.

### Statistical analysis

Statistical analyses were performed using R software (version 4.4.3; R Foundation for Statistical Computing, Vienna, Austria). Categorical variables were summarized as counts and percentages, and continuous variables as means ± standard deviations. The normality of continuous variables was assessed using the Shapiro–Wilk test, and homogeneity of variances was evaluated using Levene's test. For unadjusted comparisons among the three Kmean groups, one-way analysis of variance (ANOVA) was used for approximately normally distributed variables with homogeneous variances, followed by Tukey's honestly significant difference test for pairwise comparisons. When the assumption of homogeneity of variances was not met, Welch's ANOVA followed by the Games–Howell test was used. For non-normally distributed variables, the Kruskal–Wallis test followed by Dunn's test with Holm adjustment for multiple comparisons was performed. Specifically, anterior chamber depth, flattening effect of the anterior cornea, and torque of the posterior cornea were analyzed using one-way ANOVA, whereas all other continuous variables were analyzed using the Kruskal–Wallis test. Categorical variables were compared using the chi-square test. SIA data were analyzed using vector-based methods. Astigmatism magnitude and axis were converted into rectangular vector components (J0 and J45) using the double-angle transformation, where $\mathrm{JO}=-\frac{C}{2}\cos\;2\theta$ and $J45=-\frac{C}{2}\sin\;2\theta$, with C representing the astigmatism magnitude and θ representing the astigmatism axis. Between-group differences in these vector components were assessed using one-way multivariate analysis of variance. The assumptions of multivariate normality and homogeneity of covariance matrices were assessed before analysis. HOAs were evaluated over a standardized 6-mm analysis zone using Pentacam and iTrace measurements. Total HOA was expressed as the root mean square of the corresponding higher-order Zernike coefficients. Individual aberration components, including spherical aberration (SA), coma, and trefoil, were also analyzed. When calculated from individual Zernike coefficients, SA was represented by Z4^0^, whereas coma and trefoil were expressed as the RMS of their corresponding orthogonal Zernike components.

Multivariable linear regression analyses were performed to assess the independent associations of Kmean group with postoperative HOA and SIA outcomes. Kmean group was entered as a categorical variable, with the regular group serving as the reference category. Prespecified covariates included age, axial length (AL), white-to-white distance (WTW), and preoperative astigmatism magnitude. For postoperative HOA outcomes, the corresponding preoperative HOA value was additionally included to account for baseline differences among groups. Model fit and assumptions were assessed using R^2^, residual plots, and variance inflation factors (VIFs). Adjusted regression coefficients (*β*), 95% confidence intervals (CIs), and *P* values were reported. A *P* value < 0.05 was considered statistically significant.

## Results

A total of 162 eyes from 162 patients underwent cataract surgery during the study period. Complete data were available for 30 eyes in the flat group, 105 eyes in the regular group, and 27 eyes in the steep group (Supplementary Figure S1). The clinical characteristics of the three groups are summarized in Table 1. Regarding baseline anatomical parameters, there was a highly significant difference in the white-to-white (WTW) distance among the groups (Kruskal–Wallis test, *p* < 0.001). Specifically, the steep group exhibited a distinctly smaller WTW diameter (11.00 ± 0.31 mm) compared to the regular (11.21 ± 0.41 mm) and flat (11.53 ± 0.46 mm) groups. Other baseline clinical parameters, including age (Kruskal–Wallis test, *p* = 0.874), sex distribution (Chi-square test, *p* = 0.311), and anterior chamber depth (one-way ANOVA, *p* = 0.583), were comparable across groups. No patients required Nd:YAG capsulotomy during the study period.

**Table 1:** Baseline clinical characteristics of the three groups.

Variables	Flat	Regular	Steep	*P* value
No. eyes	30	105	27	
Sex, M, %(n)	43.3 (13)	30.5 (32)	25.9 (7)	0.311^a^
Age, years	68.7 ± 12.7	69.8 ± 11.2	69.0 ± 11.0	0.874^b^
CDVA, logMAR	0.38 ± 0.22	0.37 ± 0.22	0.37 ± 0.21	0.924^b^
Astigmatism, D	1.07 ± 0.50	0.99 ± 0.62	1.06 ± 0.63	0.718^b^
Astigmatism meridian,°	82.03 ± 61.68	98.86 ± 63.36	95.44 ± 65.77	0.406^b^
IOL type, toric, % (n)	30.0 (9)	32.4 (34)	33.3 (9)	0.959^a^
Axial length, mm	23.98 ± 1.42	23.88 ± 1.92	23.76 ± 1.84	0.103^b^
Mean keratometric astigmatism, D	0.98 ± 0.56	0.94 ± 0.57	1.03 ± 0.52	0.256^b^
Central corneal thickness, μm	552.3 ± 32.9	542.2 ± 32.0	545.2 ± 22.9	0.451^b^
Pupil diameter, mm	5.36 ± 0.73	5.23 ± 0.99	5.18 ± 0.96	0.458^b^
Anterior chamber depth, mm	3.01 ± 0.53	3.05 ± 0.48	3.13 ± 0.42	0.583^c^
White to white, mm	11.53 ± 0.46	11.21 ± 0.41	11.00 ± 0.31	< 0.001^b^

Continuous variables are presented as means ± standard deviations, and categorical variables as percentages (n).

M, male; CDVA, corrected distance visual acuity; logMAR, logarithm of the minimum angle of resolution; D, diopter.

a Result from the Chi-square test.

b Results from the Kruskal–Wallis test.

c Results from one-way analysis of variance.

### Visual acuity

CDVA improved significantly in all three groups at 3 months postoperatively (all *p* < 0.001), from preoperative values of 0.38, 0.37, and 0.37 logMAR in the flat, regular, and steep groups, respectively, to 0.07, 0.06, and 0.05 logMAR, corresponding to mean changes (*Δ*logMAR) of −0.31, −0.31, and −0.32, respectively. No significant differences in CDVA were detected between the three groups preoperatively (*p* = 0.924) or at the 1-week, 1-month, and 3-month follow-up visits (*p* = 0.390, 0.432, and 0.511, respectively) (Figure 1). Preoperative and 3-month postoperative Kmean differed significantly among the three groups (both *p* < 0.001).

**Figure 1:**
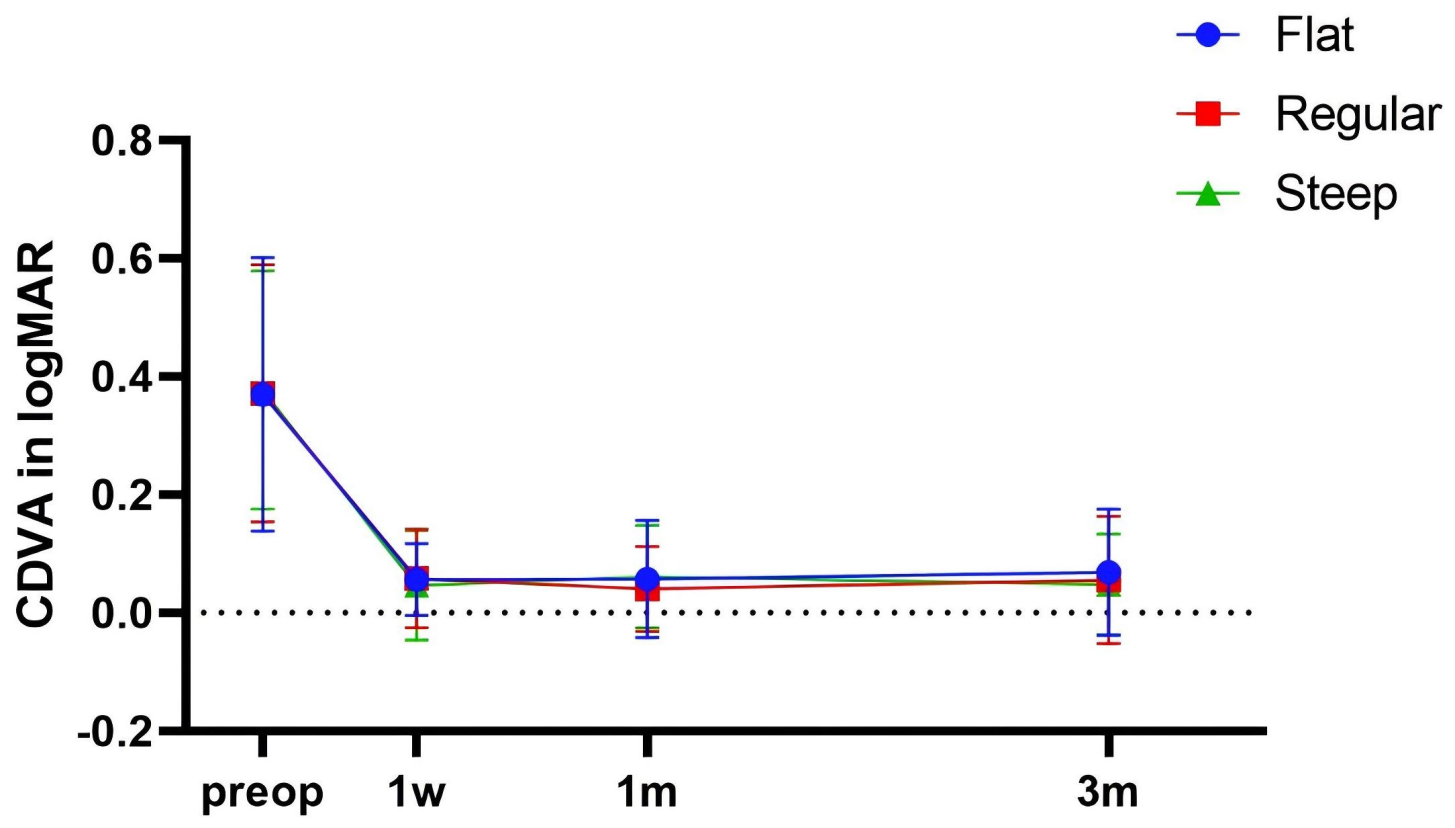
CDVA at different time points in the three groups. Statistical comparisons were performed using the Kruskal–Wallis test. Error bars represent standard deviations. CDVA = corrected distance visual acuity; logMAR = logarithm of the minimum angle of resolution; preop = preoperative.

### Corneal curvature and astigmatism

Preoperative and 3-month postoperative Kmean differed significantly among the three groups (both *P* < 0.001). Postoperatively, Kmean increased in the flat group but decreased in the other two groups, although the changes were not statistically different among groups (*p* = 0.590) (Table 2). No significant intergroup differences in anterior or posterior corneal astigmatism were observed preoperatively or at 1 and 3 months postoperatively (all *p* > 0.05) (Figure 2). At 3 months postoperatively, no significant differences were observed among the Kmean groups in any SIA-related parameter, including SIA magnitude, J0, J45, flattening effect, or torque on either the anterior or posterior corneal surface (all *P* > 0.05) (Tables 3, 4). Vector analysis demonstrated similar centroid distributions among the three groups (Figure 3). In multivariable linear regression, neither the flat group (adjusted *β* = −0.02 D; 95% CI, −0.16 to 0.12; *P* = 0.783) nor the steep group (adjusted *β* = −0.05 D; 95% CI, −0.20 to 0.10; *P* = 0.512) was significantly associated with anterior corneal SIA magnitude compared with the regular group after adjustment for age, AL, WTW, and preoperative astigmatism. The model had an R^2^ of 0.087, with all VIFs <2.5 and no major violations of regression assumptions.

**Table 2:** Changes in corneal curvature measured by pentacam preoperatively and at 3 months postoperatively.

Variables	Flat	Regular	Steep	Overall *P* value
Preoperative Kmean, D	42.16 ± 0.70	44.32 ± 0.73	46.82 ± 0.67	< 0.001
Postoperative Kmean, D	42.21 ± 0.76	44.31 ± 0.80	46.81 ± 0.59	< 0.001
Difference	0.05 ± 0.24	−0.02 ± 0.24	−0.02 ± 0.22	0.590

Statistical analysis was performed using the Kruskal–Wallis test.

F, flat; R, regular; S, steep; Kmean, mean keratometry; D, diopter; NA, not applicable.

**Figure 2:**
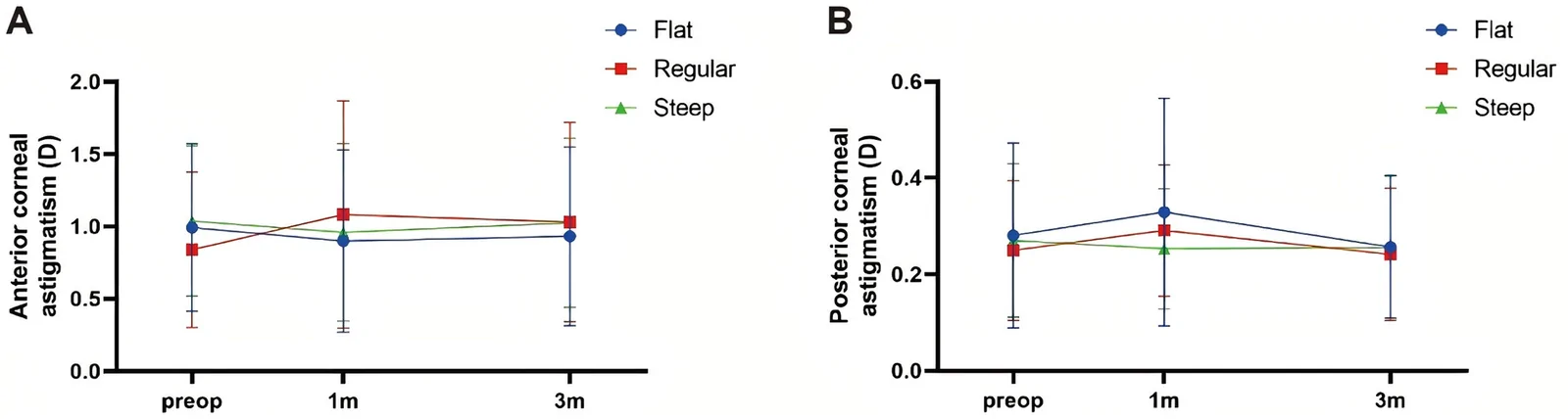
Corneal astigmatism at different time points in the three groups. **(A)** anterior corneal astigmatism. **(B)** posterior corneal astigmatism. Statistical comparisons were performed using the Kruskal–Wallis test. No significant between-group differences were observed at any time point. Preop, preoperative; D, diopter.

**Table 3:** SIA assessment results of the anterior cornea at 3 months postoperatively.

Variables	Flat	Regular	Steep	*P* value
SIA magnitude, D	0.58 ± 0.31	0.60 ± 0.32	0.54 ± 0.29	0.621^a^
J0, D	0.43 ± 0.30	0.47 ± 0.31	0.39 ± 0.28	0.488^c^
J45, D	−0.05 ± 0.24	0.06 ± 0.25	0.02 ± 0.23	0.371^c^
Centroid SIA, D @ axis	0.43 @ 94.3	0.50 @ 93.6	0.41 @ 88.7	0.571^c^
Centroid SIA, magnitude @ axis	0.43 @ 94.3	0.50 @ 93.6	0.41 @ 88.7	NA
Flattening effect, D	0.24 ± 0.51	0.00 ± 0.53	0.15 ± 0.44	0.083^b^
Torque, D	0.04 ± 0.38	−0.02 ± 0.43	0.09 ± 0.41	0.709^a^
Absolute torque, D	0.28 ± 0.26	0.32 ± 0.24	0.34 ± 0.25	0.576^a^

NA, not applicable; postop, postoperative; SIA, surgically induced astigmatism; D, diopter.

a Results from the Kruskal–Wallis test.

b Results from one-way analysis of variance.

c Results from one-way multivariate analysis of variance.

**Table 4:** SIA assessment results of the posterior cornea at 3 months postoperatively.

Variables	Flat	Regular	Steep	*P* value
SIA magnitude, D	0.13 ± 0.07	0.11 ± 0.06	0.12 ± 0.06	0.302^a^
J0, D	−0.01 ± 0.06	0.00 ± 0.06	0.01 ± 0.07	0.641^c^
J45, D	−0.02 ± 0.06	0.02 ± 0.06	0.00 ± 0.07	0.553^c^
Centroid SIA, D @ axis	0.03 @ 128.1	0.05 @ 18.5	0.04 @ 1.4	0.674^c^
Centroid SIA, magnitude @ axis	0.03 @ 128.1	0.05 @ 18.5	0.04 @ 1.4	NA
Flattening effect, D	0.04 ± 0.10	0.04 ± 0.09	0.03 ± 0.10	0.656^a^
Torque, D	0.02 ± 0.07	0.03 ± 0.07	0.03 ± 0.08	0.500^b^
Absolute torque, D	0.06 ± 0.05	0.06 ± 0.05	0.06 ± 0.06	0.774^a^

NA, not applicable; postop, postoperative; SIA, surgically induced astigmatism; D, diopter.

a Results from the Kruskal–Wallis test.

b Results from one-way analysis of variance.

c Results from one-way multivariate analysis of variance.

**Figure 3:**
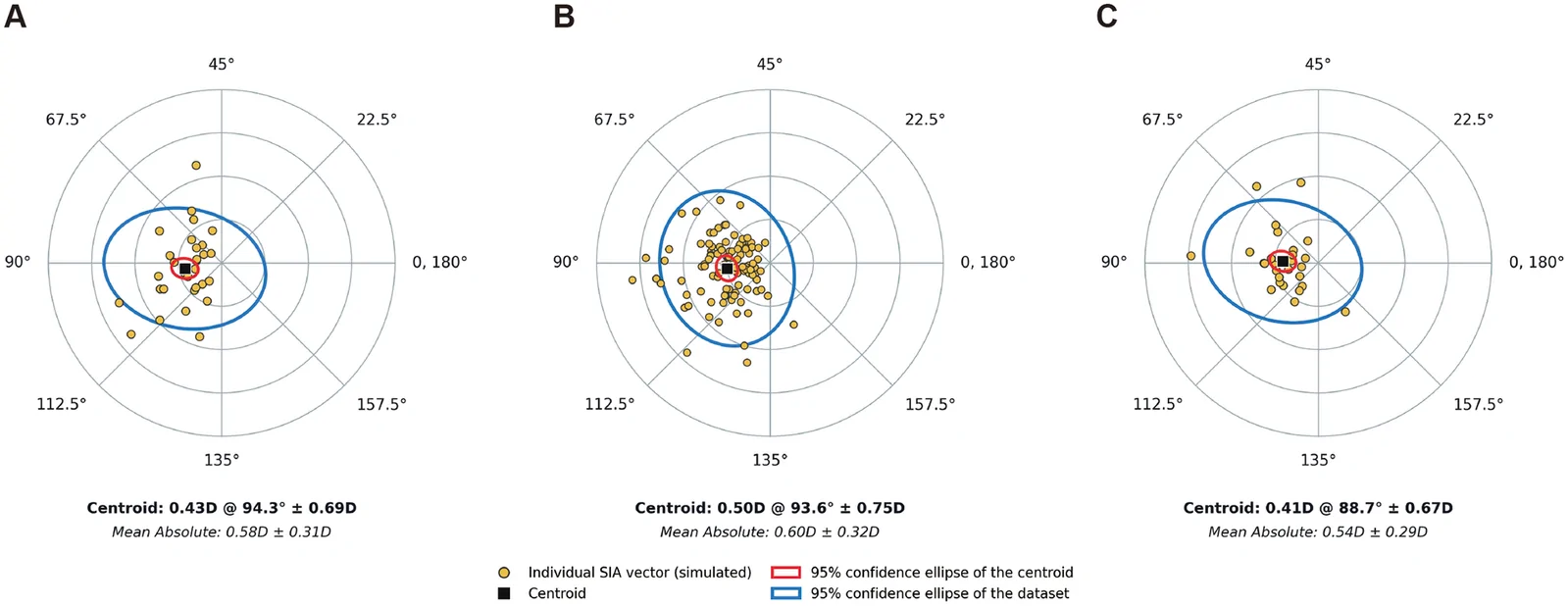
Double-angle plots of SIA vectors of the anterior cornea at 3 months postoperatively. **(A)** flat group. **(B)** regular group. **(C)** steep group. Each ring = 0.50 D. SIA, surgically induced astigmatism; D, diopter.

### Corneal HOAs

Preoperatively, the flat group had significantly lower anterior corneal HOA RMS than the steep group (Hodges–Lehmann difference, −0.17 μm; 95% CI, −0.29 to −0.06; *P*adj = 0.009) and lower posterior corneal HOA RMS (−0.05 μm; 95% CI, −0.08 to −0.02; *P*adj < 0.001). At 3 months postoperatively, the flat group continued to show lower anterior corneal HOA RMS than the steep group (−0.18 μm; 95% CI, −0.29 to −0.07; *P*adj = 0.005) and lower posterior corneal HOA RMS (−0.03 μm; 95% CI, −0.06 to −0.01; *P*adj = 0.018). However, changes from baseline in anterior and posterior corneal HOA RMS did not differ significantly among the three groups (all *P* > 0.05) (Table 5), confirming no differential impact of surgery on total HOAs across Kmean groups.

**Table 5:** Changes in total HOAs measured by pentacam over the 6 mm analytical zone preoperatively and at 3 months postoperatively.

Variables	Flat	Regular	Steep	Omnibus *P* value	F–R, *P*adj	F–S, *P*adj	R–S, *P*adj
Total corneal HOA RMS, μm							
Preoperative	0.61 ± 0.21	0.69 ± 0.25	0.77 ± 0.26	0.067	NA	NA	NA
Postoperative	0.67 ± 0.23	0.79 ± 0.39	0.83 ± 0.26	0.052	NA	NA	NA
Difference	0.07 ± 0.18	0.10 ± 0.32	0.06 ± 0.14	0.909	NA	NA	NA
Anterior corneal HOA RMS, μm							
Preoperative	0.55 ± 0.14	0.63 ± 0.20	0.72 ± 0.22	**0**.**007**	0.227	**0.009**	0.088
Postoperative	0.60 ± 0.16	0.72 ± 0.32	0.78 ± 0.24	**0**.**007**	0.121	**0.005**	0.189
Difference	0.06 ± 0.13	0.09 ± 0.26	0.06 ± 0.12	0.996	NA	NA	NA
Posterior corneal HOA RMS, μm							
Preoperative	0.19 ± 0.08	0.20 ± 0.04	0.24 ± 0.08	**< 0.001**	**< 0.001**	**< 0.001**	**< 0.001**
Postoperative	0.21 ± 0.05	0.21 ± 0.05	0.24 ± 0.05	**0**.**010**	1.000	**0.018**	**0.027**
Difference	0.02 ± 0.08	0.01 ± 0.06	−0.01 ± 0.10	0.710	NA	NA	NA

Statistical analysis was performed using the Kruskal–Wallis test followed by Dunn's test with Holm adjustment for multiple comparisons.

F, flat; R, regular; S, steep; NA, not applicable; HOAs, higher-order aberrations; RMS, root mean square.

Preoperative iTrace measurements showed that between-group differences in HOAs were primarily attributable to SA, which was significantly lower in the flat group than in the steep group (−0.09 μm; 95% CI, −0.15 to −0.03; *P*adj = 0.001). At 3 months postoperatively, neither total HOA RMS nor any individual aberration component differed significantly among the three groups. For changes from baseline, only the increase in trefoil was significantly greater in the flat group than in the regular group (0.10 μm; 95% CI, 0.02 to 0.18; *P*adj = 0.042) after adjustment for multiple comparisons, whereas changes in the other aberration components, including SA, were not statistically significant (Table 6). At a 6.0-mm pupil, this 0.10-*μ*m increase is minimal and unlikely to cause perceptible visual degradation.

**Table 6:** Changes in HOAs of the total cornea measured by iTrace over the 6 mm analytical zone preoperatively and at 3 months postoperatively.

	Flat	Regular	Steep	Omnibus *P* value	F–R, *P*adj	F–S, *P*adj	R–S, *P*adj
Total HOA RMS, μm							
Preoperative	0.44 ± 0.11	0.53 ± 0.17	0.57 ± 0.14	**0**.**004**	**0.036**	**0.007**	0.382
Postoperative	0.56 ± 0.22	0.57 ± 0.18	0.62 ± 0.15	0.100	NA	NA	NA
Difference	0.13 ± 0.24	0.03 ± 0.18	0.04 ± 0.14	0.086	NA	NA	NA
Spherical aberration RMS, μm							
Preoperative	0.21 ± 0.10	0.25 ± 0.10	0.30 ± 0.09	**0**.**003**	0.498	**0.001**	**0.021**
Postoperative	0.23 ± 0.09	0.24 ± 0.09	0.27 ± 0.10	0.490	NA	NA	NA
Difference	0.01 ± 0.10	−0.01 ± 0.09	−0.04 ± 0.09	0.121	NA	NA	NA
Coma RMS, μm							
Preoperative	0.21 ± 0.10	0.25 ± 0.17	0.29 ± 0.15	0.170	NA	NA	NA
Postoperative	0.24 ± 0.16	0.24 ± 0.17	0.30 ± 0.15	0.097	NA	NA	NA
Difference	0.02 ± 0.16	−0.02 ± 0.14	0.01 ± 0.12	0.182	NA	NA	NA
Trefoil RMS, μm							
Preoperative	0.21 ± 0.08	0.27 ± 0.15	0.29 ± 0.15	0.156	NA	NA	NA
Postoperative	0.31 ± 0.16	0.29 ± 0.16	0.29 ± 0.14	0.512	NA	NA	NA
Difference	0.11 ± 0.18	0.01 ± 0.21	0.01 ± 0.16	**0**.**039**	**0.042**	0.118	0.967

Statistical analysis was performed using the Kruskal–Wallis test followed by Dunn's test with Holm adjustment for multiple comparisons.

F, flat; R, regular; S, steep; NA, not applicable; HOAs, higher-order aberrations; RMS, root mean square.

In multivariable linear regression analysis, Kmean group was not independently associated with postoperative total corneal HOA RMS measured by Pentacam after adjustment for age, AL, WTW, preoperative astigmatism, and preoperative HOA RMS. Compared with the regular group, the adjusted *β* was −0.03 μm (95% CI, −0.12 to 0.06; *P* = 0.510) for the flat group and 0.04 μm (95% CI, −0.05 to 0.13; *P* = 0.393) for the steep group. The model had an R^2^ of 0.423, with all VIFs <2.5 and no major violations of regression assumptions. Overall, after adjustment for prespecified covariates, Kmean group was not independently associated with postoperative SIA magnitude or total corneal HOA RMS.

## Discussion

This study compared visual and refractive outcomes among patients with flat, regular, and steep corneal meridians undergoing phacoemulsification with IOL implantation for age-related cataract. Surgical planning and patient allocation were based on preoperative corneal astigmatism, which was comparable across the three groups. In line with routine clinical practice, patients with lower astigmatism generally received monofocal IOLs, whereas those with higher astigmatism were implanted with toric IOLs. Because IOL selection primarily reflects internal optical correction, it was unlikely to have significantly affected the subsequent analyses of corneal astigmatism or HOAs. Accordingly, the grouping strategy employed mirrors standard clinical decision-making and enhances the generalizability of the findings to broader patient populations and real-world treatment scenarios.

In terms of baseline characteristics, a significant difference was observed in white-to-white distance, with the steep group exhibiting a smaller corneal diameter. This finding aligns with the notion that a reduced corneal diameter can lead to corneal warping and increased curvature (20). Other baseline characteristics were comparable across groups.

Regarding postoperative changes in corneal curvature, previous studies have reported that a 2.75-mm corneal incision exerts a slightly greater flattening than steepening effect on corneal shape (21). Our study refined this observation by stratifying eyes according to preoperative corneal curvature. We found that Kmean increased in the flat group, suggesting a predominant steepening effect, whereas Kmean decreased in the regular and steep groups, indicating a flattening effect.

Despite these morphological variations, no clinically meaningful differences were observed in refractive or visual outcomes. Postoperative CDVA, astigmatism, and SIA were comparable among the three groups. Although prior research suggested that corneal diameter may influence postoperative astigmatism, with smaller white-to-white distances associated with greater SIA (10), this relationship was not observed in our cohort.

Patients in the flat keratometry group exhibited lower preoperative HOAs in both the anterior and posterior cornea compared with those in the steep group, suggesting that steeper corneas are inherently more aberrated. This difference persisted at 3 months postoperatively. Although the increase in HOAs from baseline was slightly greater in the flat group, the between-group differences were not statistically significant, indicating that cataract surgery itself does not substantially alter total HOAs as a function of preoperative keratometry.

The shift in the source of aberrations warrants closer consideration. Previous studies have reported that all corneal HOA components (22), including SA (23) and trefoil (24), tend to increase after cataract surgery. In our cohort, the flat group demonstrated lower preoperative HOAs across all components, with SA showing the most pronounced difference. This finding is consistent with the optical principle that steeper corneas amplify SA through greater peripheral light-ray deviation (12). Postoperatively, these intergroup differences narrowed, largely because HOAs increased more in the flat group. In particular, the increase in trefoil aberration was significantly greater in the flat group compared to the regular group. Concurrently, SA showed a slight trend of increasing in the flat group but decreasing in the regular and steep groups, although this difference did not reach statistical significance. These findings suggest that flatter corneas exhibit a statistically significant increase in trefoil aberration compared to regular corneas (0.10 μm; 95% CI, 0.02 to 0.18; *P*adj = 0.042), highlighting that specific localized aberrations (such as trefoil) rather than overall HOAs are more sensitive to preoperative curvature variations. From a clinical perspective, these findings may provide useful context for considerations regarding surgical planning and IOL selection. Eyes with steep corneas demonstrated higher intrinsic SA and total HOAs both preoperatively and postoperatively, suggesting that these patients may possess a more optically vulnerable corneal profile. Although our cohort only included monofocal IOLs, we speculate that implanting diffractive multifocal IOLs in these eyes could theoretically exacerbate dysphotopsia, a premise that warrants dedicated clinical validation. Conversely, eyes with flat corneas appeared to exhibit a greater tendency for incision-induced trefoil compared to regular corneas, with a non-significant trend towards increased SA. This observation suggests that surgical technique may play a greater role in preserving postoperative optical quality in flatter corneas. Additionally, given that a standardized 3.0-mm incision was utilized in this study, minimizing incision-related biomechanical disruption through smaller clear corneal incisions (e.g., 2.2 mm or less) or shorter tunnel construction may help reduce the induction of higher-order aberrations in flatter corneas, which warrants further investigation in future studies.

This real-world prospective study demonstrates that cataract surgery yields effective and consistent refractive and visual outcomes in eyes with both flat and steep keratometry. Steeper corneas exhibit higher intrinsic HOAs, whereas flatter corneas may exhibit a greater tendency for trefoil induction compared to regular corneas, warranting further investigation in larger cohorts. These findings suggest that preoperative corneal curvature characteristics may be considered in refractive planning, as well as in informing surgical techniques and premium IOL selection to maximize postoperative visual quality.

### Limitations

This study has several limitations. First, the sample sizes differed among groups because extremely flat and steep corneas are relatively uncommon in the general population, which is a typical feature of real-world clinical studies. Consequently, tests for normality and homogeneity of variances (such as Shapiro–Wilk and Levene tests) possessed limited statistical power, and the study may be underpowered for detecting subtle yet clinically relevant differences in surgically induced astigmatism (SIA) and higher-order aberrations (HOAs). Larger multicenter studies are needed to further validate these findings. Second, the follow-up period was limited to 3 months. Because corneal biomechanical remodeling and stabilization of astigmatism and higher-order aberrations may continue for 6 months or longer after surgery, longer-term follow-up is warranted. Third, we did not evaluate subjective visual symptoms, such as patient-reported dysphotopsia, and the clinical cohort was limited to patients receiving monofocal or toric IOLs. Therefore, the specific interaction between extreme corneal morphology and multifocal optics on subjective visual quality warrants further investigation. Fourth, our cohort was restricted to right eyes only, and all procedures were performed by a single surgeon; although this design minimizes confounding variables and ensures surgical consistency, it may limit the generalizability of our findings.

## Data Availability

The raw data supporting the conclusions of this article will be made available by the authors, without undue reservation.
