# Supplementary material for: Impact of preoperative mean keratometry on corneal higher-order aberrations and optical quality following cataract surgery
Source: Front Med (Lausanne). 2026 Oct 13;13:1934290. doi: 10.3389/fmed.2026.1934290 (PMC13710270; doi:10.3389/fmed.2026.1934290)
Supplement: Supplementary file 1 [file Table1.docx]

**Supplementary Figure 1.** Flow diagram of patient screening, inclusion, and exclusion.

The flowchart illustrates the step-by-step selection process of the study cohort in accordance with routine clinical practice. A total of 225 patients were initially assessed for eligibility, of which 63 were excluded due to preoperative conditions, intraoperative complications, or loss to follow-up. The final analysis included 162 eyes, which were stratified into flat (n = 30), regular (n = 105), and steep (n = 27) groups based on their preoperative mean keratometry (Kmean).

**Supplementary Figure 2.** Distribution of preoperative mean keratometry (Kmean) across the entire study cohort (n = 162 eyes). Patients were stratified into flat (≤43.0D), regular (43.0–46.0D), and steep (≥46.0D) groups based on Kmean values.

Abbreviation: D = diopter.
